# Cross-domain antimicrobial resistance in poultry farming: A One Health assessment of antimicrobial use and multidrug resistance in Kiambu County, Kenya

**DOI:** 10.14202/vetworld.2026.1-14

**Published:** 2025-01-06

**Authors:** Ann Kangai Munene, Peter Muiruri Mwangi, Lilly Caroline Bebora, Christine Minoo Mbindyo, John Muthini Maingi

**Affiliations:** 1 Department of Biochemistry, Microbiology, and Biotechnology, Kenyatta University, Nairobi, Kenya; 2 Department of Veterinary Pathology, Microbiology, and Parasitology, University of Nairobi, Nairobi, Kenya; 3 International Center of Insect Physiology and Ecology, Nairobi, Kenya

**Keywords:** Antimicrobial use, antimicrobial resistance, poultry farming, *Escherichia coli*, *Enterococcus*, One Health, Kenya

## Abstract

**Background and Aim:**

Antimicrobial resistance (AMR) has emerged as a major One Health threat driven by inappropriate antimicrobial use (AMU) in humans, animals, and the environment. Poultry production is recognized as a key reservoir of antimicrobial-resistant bacteria, yet few studies in Kenya examine AMU and AMR across interconnected human–animal–environment domains. This study assessed AMU patterns among poultry farmers in Kiambu County and characterized phenotypic resistance in *Escherichia coli* and *Enterococcus* spp. isolated from humans, chickens, and chicken environments.

**Materials and Methods:**

A cross-sectional study was conducted from June to September 2024, involving 102 poultry farms. Farm demographics and AMU data were collected using a semi-structured questionnaire. Archived *E. coli* (n = 92) and *Enterococcus* spp. (n = 101) isolates from chicken handlers’ hands, chickens, and environmental samples were subjected to antimicrobial susceptibility testing using the Kirby–Bauer method per Clinical and Laboratory Standards Institute (CLSI) 2024 guidelines. Descriptive and inferential statistics, including logistic regression with false discovery rate correction, were used to assess associations between AMU and phenotypic resistance.

**Results:**

Macrolides (69%), tetracyclines (48%), and sulfonamides (21%) were the most commonly used antimicrobials; 7% of farms reported colistin use. Among *E. coli* isolates, resistance was highest to ampicillin (77%), tetracycline (72%), and trimethoprim–sulfamethoxazole (49%), with 35% exhibiting multidrug resistance (MDR). No carbapenem resistance was detected. *Enterococcus* isolates showed high erythromycin resistance (61%) and moderate ciprofloxacin resistance (26%), with 6.9% exhibiting MDR; no vancomycin-resistant enterococci (VRE) were observed. Penicillin use strongly predicted ampicillin resistance in both organisms, whereas sulfonamide use was associated with reduced trimethoprim–sulfamethoxazole resistance. Macrolide use did not correlate with erythromycin resistance.

**Conclusion:**

High AMU in poultry farming, particularly of macrolides, tetracyclines, and sulfonamides, has created significant selection pressure, contributing to MDR emergence across One Health interfaces. Detection of resistance in humans, poultry, and shared environments underscores the bidirectional risk of AMR transmission. Strengthened antimicrobial stewardship, regulation of critically important antimicrobials, and enhanced farm hygiene are essential to mitigate AMR. These findings directly support Kenya’s Vision 2030 and SDGs targeting health, responsible production, and environmental protection.

## INTRODUCTION

Antimicrobial resistance (AMR) has emerged as a growing global threat in which microorganisms, including bacteria, fungi, and parasites, no longer respond to antimicrobial agents to which they were once susceptible, yet continue to survive and proliferate in their presence [1, 2]. The irrational and widespread use of antimicrobials across human health, livestock production, and crop agriculture has accelerated this growing crisis, undermining the One Health framework that links human, animal, and environmental health [1, 3]. Beyond resistance genes associated with clinical infections, there is increasing recognition that pathogenic, commensal, and environmental bacteria all play essential roles in driving the emergence and dissemination of AMR [4]. Notably, approximately 70% of antimicrobials classified for human use have been reported in veterinary applications [1]. These agents are routinely administered in food-producing animals for disease treatment and prevention, as well as for non-therapeutic purposes such as growth promotion and improved production efficiency [5–7].

In Kenya, antimicrobial use (AMU) for disease management and productivity enhancement in poultry has intensified across both large- and small-scale farming operations [8, 9]. As a result, extensive AMU and the persistence of antimicrobial residues in humans, animals, and the environment create strong selection pressure that fosters the emergence and spread of resistant commensal and pathogenic bacteria [7, 10]. Humans may acquire antimicrobial-resistant bacteria and resistance genes from animals through direct or indirect contact with livestock and by consuming contaminated animal-derived food products [6].

Despite Kenya’s recognition of AMR as a national priority and the establishment of a One Health–aligned National Action Plan to guide AMR prevention and containment efforts [11], there remains a critical shortage of empirical data characterizing AMR at the human–animal–environment interface within local livestock systems. Existing national and regional reports primarily emphasize surveillance gaps and implementation challenges but provide limited farm-level evidence on how AMU in poultry production contributes to resistance patterns among bacteria circulating among humans, animals, and shared environments. Furthermore, few studies in Kenya have simultaneously examined AMU practices alongside phenotypic resistance profiles of *Escherichia coli* and *Enterococcus* spp., key commensal indicator organisms recommended for integrated AMR monitoring. The scarcity of context-specific One Health studies constrains the ability to evaluate the effectiveness of current policy interventions, identify high-risk practices, and design targeted mitigation strategies for poultry farming communities. This knowledge gap underscores the urgent need for localized, cross-domain assessments of AMU and AMR to inform Kenya’s ongoing AMR policy implementation and strengthening efforts [11].

In line with Kenya’s National Action Plan on AMR, which emphasizes integrated One Health surveillance and evidence-driven policy action [11], this study aimed to generate farm-level data on AMU and AMR in poultry production systems in Kiambu County. Specifically, the study sought to (i) assess AMU patterns among poultry farmers and (ii) determine the phenotypic resistance profiles of *E. coli* and *Enterococcus* isolates from chickens, chicken handlers, and poultry environments. By examining AMU and AMR concurrently across interconnected domains, the study provides essential evidence to support national efforts to improve antimicrobial stewardship, strengthen surveillance, and guide risk-reduction strategies within Kenya’s poultry sector. Findings will contribute to Kenya’s Vision 2030 and the Sustainable Development Goals (SDGs), particularly SDG3 (Good health and well-being), SDG12 (Responsible product consumption and production), and SDG15 (Safe environment and ecosystems).

## MATERIALS AND METHODS

### Ethical approval

Ethical approval for this study was obtained in accordance with One Health ethical standards governing research involving humans, animals, and shared environments. The Kenyatta University Ethics Review Committee (KU-ERC) approved the research protocol under approval number PKU/2895/2019, and the National Commission for Science, Technology and Innovation (NACOSTI) issued a research permit (Ref No.: 877111; License No.: NACOSTI/P/24/34222).

Access to poultry farms was authorized by the Sub-County Veterinary Officers and Livestock Production Officers of Kiambu County, Kenya. All participating poultry farmers and chicken handlers provided written informed consent before data collection. Participants were informed of the study objectives, confidentiality safeguards, and their right to withdraw at any time without penalty.

Archived *E. coli* and *Enterococcus* spp. isolates generated in a previous study under the same ethical clearance were used for this investigation [12]. No live animals were handled, restrained, or euthanized. All laboratory procedures were performed under BSL-2 conditions and followed the Clinical and Laboratory Standards Institute (CLSI M100-ED34, February 2024, Volume 44, Number 5) guidelines [13]. Experimental procedures complied with the Kenya Veterinary Board Code of Conduct, the World Organization for Animal Health (WOAH/OIE) animal welfare standards, and the Declaration of Helsinki (2013) for research involving human participants.

### Study period and location

**Figure 1:**
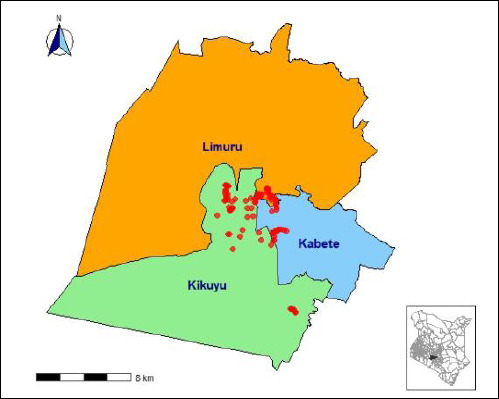
Map of three subcounties (Kabete, Kikuyu, and Limuru) where bacterial isolates were collected. The inset shows the location of Kiambu County in Kenya.

The study was conducted from June to September 2024 in Kiambu County, Kenya, specifically in the Kabete, Kikuyu, and Limuru sub-counties (1.0314° S, 36.8681° E; Figure 1). The isolates analyzed in this investigation were obtained during a previous cross-sectional prevalence study of poultry farms in the region [12].

### Sample size and isolate selection

A total of 102 poultry farms contributed archived bacterial isolates representing the One Health domains of humans, animals, and the environment. For *E. coli*, 92 isolates were included: 13 from chicken handlers’ hands, 25 from chicken cloacae, 27 from chicken house floor samples, and 27 from the exterior surroundings of chicken houses.

For *Enterococcus* spp., 101 isolates were analyzed: 25 from chicken handlers’ hands, 25 from chicken cloacae, 26 from chicken house floors, and 25 from exterior environments. These isolates collectively represented human–animal–environmental microbial interactions across poultry production systems.

### Antimicrobial susceptibility testing (AST)

#### Preparation and revival of bacterial isolates

**Figure 2:**
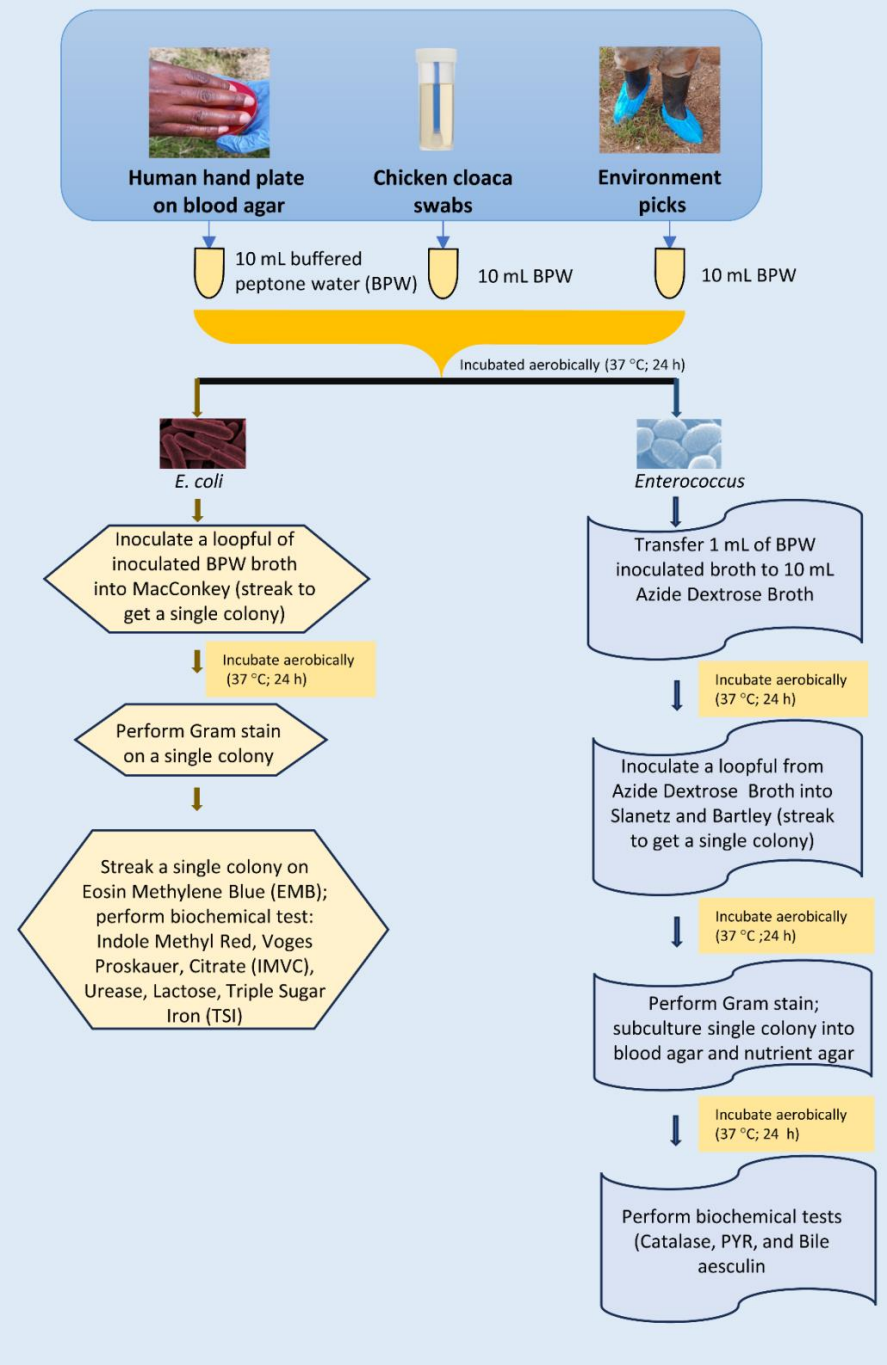
Laboratory isolation flow chart of *Escherichia coli* and *Enterococcus*.

Previously isolated, as shown in Figure 2, and cryopreserved pure cultures of *E. coli* and *Enterococcus* spp. (stored in 20% tryptone soya broth at –20°C for six months and thawed once) were revived and subcultured onto MacConkey agar (*E. coli*) and blood agar (*Enterococcus* spp.). These isolates originated from chickens, chicken handlers’ hands, and poultry environmental samples.

#### Kirby–Bauer disk diffusion procedure

AST was performed according to CLSI guidelines [14] using the Kirby–Bauer disk diffusion method on Mueller–Hinton agar. Briefly, 3–5 colonies from each overnight culture were emulsified in sterile saline and adjusted to a 0.5 McFarland turbidity standard. Sterile swabs were dipped into each suspension, rotated to remove excess liquid, and streaked onto plates to ensure confluent growth. All plate dimensions, incubation conditions, and interpretive criteria complied with CLSI recommendations. Each test was performed in duplicate and measured with a digital Vernier caliper. Complete and intermediate resistance profiles were interpreted according to CLSI criteria [15].

#### AST for *E. coli*

For *E. coli*, 150-mm Mueller–Hinton agar plates were used to accommodate nine antimicrobial disks without zone overlap. A total of 92 isolates were tested against the following antimicrobial classes and disk potencies:

- Cephems: ceftriaxone, 30 μg
- Penicillins: ampicillin, 10 μg
- Aminoglycosides: gentamicin, 10 μg
- Fluoroquinolones: ciprofloxacin, 5 μg
- Quinolones: nalidixic acid, 30 μg
- Phenicols: chloramphenicol, 30 μg
- Tetracyclines: tetracycline, 30 μg
- Carbapenems: ertapenem, 10 μg
- Folate pathway antagonists: trimethoprim–sulfamethoxazole, 1.25/23.75 μg

Plates were incubated at 37°C for 16–18 h. Ertapenem served as a surveillance marker for carbapenem resistance, while ceftriaxone represented a clinically relevant human therapeutic. Lot numbers and expiration dates for all disks are presented in Supplementary Table 1.

### AST for *Enterococcus*

For *Enterococcus*, 100-mm plates containing five antimicrobial disks were used; vancomycin was tested separately to avoid zone overlap. A total of 101 isolates were evaluated against:

- Fluoroquinolones: ciprofloxacin, 5 μg
- Penicillins: ampicillin, 10 μg
- Tetracyclines: tetracycline, 30 μg
- Macrolides: erythromycin, 15 μg
- Oxazolidinones: linezolid, 30 μg
- Glycopeptides: vancomycin, 30 μg

Plates were incubated at 37°C for 16–18 h, except vancomycin plates, which were incubated for 24 h. Disk details are provided in Supplementary Table 1.

### Quality control (QC) procedures

All inhibition zone diameters were interpreted in accordance with CLSI M100-Ed34 guidelines [14]. QC was ensured by including *Staphylococcus aureus* American Type Culture Collection (ATCC) 25923 (for *Enterococcus*) and *E. coli* ATCC 25922 (for *E. coli*) in all assays. QC results are provided in Table S2.

### Farm demographics and AMU data collection

Farm-level information was collected using a semi-structured questionnaire, pretested for face validity among a subset of farmers. Respondents reported antimicrobial products used in poultry production and presented any available drug containers or sachets for verification. Data on AMU and farm characteristics were recorded, and triangulation across respondents was used to confirm reliability.

### Statistical analysis

Descriptive statistics were generated in R version 4.4.2 [16] using the dplyr package [17] to compute counts and percentages of AMU and AMR. Inferential analyses employed:

- scipy.stats for Fisher’s exact tests
- statsmodels for logistic regression
- statsmodels.stats.multitest for false discovery rate (FDR) correction [18, 19]

Data visualizations were produced in Python 3.11.6 using seaborn [20] and matplotlib [21]. Multiple comparisons were adjusted using the Benjamini–Hochberg FDR method.

## RESULTS

### Antimicrobial susceptibility patterns of *E. coli*

#### Overall resistance profiles

**Figure 3:**
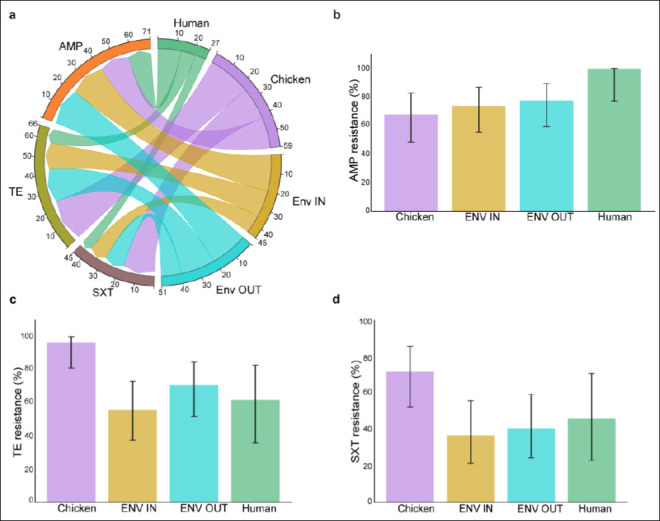
Susceptibility patterns of *Escherichia coli* isolates. (a) Comparison of *E. coli* resistance patterns across sample sources. (b–d) Resistance percentages with 95% confidence intervals for *E. coli* isolates across the One Health variables (Chicken handlers’ hands, chicken, and chicken environs). AMP = Ampicillin, TE = Tetracycline, SXT = Trimethoprim–sulfamethoxazole, ENV IN = Inside environment, ENV OUT = Outside environment.

Among the 92 *E. coli* isolates tested, high resistance levels were observed across key antimicrobial classes. Resistance to the β-lactam ampicillin was 77.2% (71/92), while tetracycline resistance was 71.7% (66/92). Additionally, trimethoprim–sulfamethoxazole resistance was 48.9% (45/92) (Figure 3a). No resistance was detected to ciprofloxacin, ceftriaxone, ertapenem, gentamicin, nalidixic acid, or chloramphenicol in any sample source, although the upper 95% confidence interval (CI) limits reached 22.8% in human isolates and 12–13% in poultry and environmental isolates (Table 1).

**Table 1:** Resistance proportions (%) and 95% confidence intervals (CIs) for *E. coli* isolates across One Health sample sources.

Sample Source	Antibiotic	Resistant n/N	% Resistant	95% CI
Chicken	SXT	18/25	72	52.4–85.7
Chicken	TE	24/25	96	80.5–99.3
Chicken	AMP	17/25	68	48.4–82.8
Chicken	CIP, CRO, ETP, CN, NA, C	0/25	0	0–13.3
Env IN	SXT	10/27	37	21.5–55.8
Env IN	TE	15/27	55.6	37.3–72.4
Env IN	AMP	20/27	74.1	55.3–86.8
Env IN	CIP, CRO, ETP, CN, NA, C	0/27	0	0–12.5
Env OUT	SXT	11/27	40.7	24.5–59.3
Env OUT	TE	19/27	70.4	51.5–84.1
Env OUT	AMP	21/27	77.8	59.2–89.4
Env OUT	CIP, CRO, ETP, CN, NA, C	0/27	0	0–12.5
Human	SXT	6/13	46.2	23.2–70.9
Human	TE	8/13	61.5	35.5–82.3
Human	AMP	13/13	100	77.2–100
Human	CIP, CRO, ETP, CN, NA, C	0/13	0	0–22.8

n/N = number of resistant isolates / total isolates tested. 95% CI = 95% Confidence interval of proportion. AMP = Ampicillin, C = Chloramphenicol, CIP = Ciprofloxacin, CN = Gentamicin, CRO = Ceftriaxone, ETP = Ertapenem, NA = Nalidixic acid, SXT = Trimethoprim–sulfamethoxazole, TE = Tetracycline.

#### Resistance patterns by One Health source

Resistance varied across human, animal, and environmental domains.

- Chicken isolates displayed extremely high resistance to tetracycline (96%, 95% CI: 80.5–99.3) and trimethoprim–sulfamethoxazole (72%, 95% CI: 52.4–85.7) (Figures 3c and 3d).
- Environmental isolates also demonstrated substantial resistance, with ampicillin resistance of 74.1% (95% CI: 55.3–86.8) indoors and 77.8% (95% CI: 59.2–89.4) outdoors (Figure 3b).
- Human hand isolates showed the highest ampicillin resistance, with 100% (13/13) resistance (95% CI: 77.2–100).

#### Multidrug resistance (MDR)

Approximately 35% (32/92) of *E. coli* isolates were classified as MDR, defined as resistance to three or more antimicrobial classes. The most prevalent MDR pattern was ampicillin + tetracycline + trimethoprim–sulfamethoxazole, reflecting the dominant resistance trends observed across the One Health sampling framework.

### Antimicrobial susceptibility patterns of *E. coli* isolates

#### Overall resistance trends

Among the 92 *E. coli* isolates tested, high levels of resistance were observed to β-lactams, tetracyclines, and folate pathway inhibitors. Specifically, resistance to ampicillin was 77.2% (71/92), to tetracycline 71.7% (66/92), and to trimethoprim–sulfamethoxazole 48.9% (45/92) (Figure 3a). No resistance to ciprofloxacin, ceftriaxone, ertapenem, gentamicin, nalidixic acid, or chloramphenicol was detected across any source; however, the upper CI reached 22.8% for human isolates and 12–13% for chicken and environmental samples (Table 1).

#### Source-specific resistance patterns

Resistance varied across One Health sources. Among chicken isolates, tetracycline resistance was extremely high at 96% (95% CI: 80.5–99.3), and trimethoprim–sulfamethoxazole resistance reached 72% (95% CI: 52.4–85.7) (Figures 3c and 3d; Table 1). In environmental samples, ampicillin resistance ranged from 74.1% (95% CI: 55.3–86.8) in indoor environments to 77.8% (95% CI: 59.2–89.4) in outdoor environments (Figure 2b). All 13 isolates obtained from chicken handlers’ hands showed 100% resistance to ampicillin (95% CI: 77.2–100).

#### MDR

Approximately 35% (32/92) of isolates met the criteria for MDR, defined as resistance to three or more antimicrobial classes. The most common MDR profile included resistance to ampicillin, tetracycline, and trimethoprim–sulfamethoxazole.

### Antimicrobial susceptibility patterns of *Enterococcus* isolates

#### Overall resistance trends

**Figure 4:**
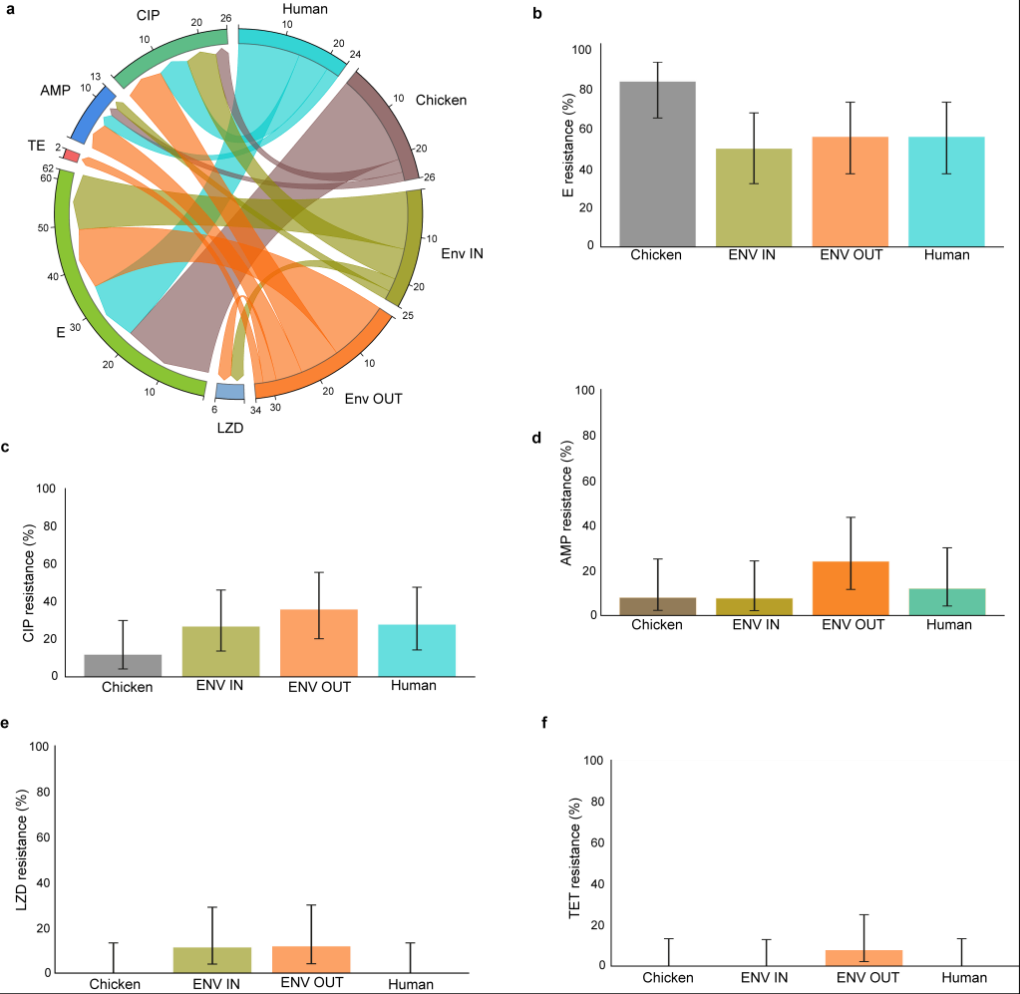
Susceptibility patterns of *Enterococcus* isolates. (a) Comparison of *Enterococcus* resistance patterns across One Health sources: chicken handlers’ hands, chicken, and chicken environs. (b–f) Resistance percentages with 95% confidence intervals for *Enterococcus* isolates. AMP = Ampicillin, CIP = Ciprofloxacin, E = Erythromycin, LZD = Linezolid, TE = Tetracycline.

Among the 101 *Enterococcus* isolates tested, erythromycin had the highest overall resistance at 61.4% (62/101), followed by ciprofloxacin (25.7%; 26/101), ampicillin (12.9%; 13/101), linezolid (5.9%; 6/101), and tetracycline (2.0%; 2/101) (Figure 4a). No resistance to vancomycin, linezolid, or tetracycline was detected, although the upper 95% CI limits suggested the potential for low-level resistance (0%–13%) (Table 2).

**Table 2:** Proportions of resistance (%) and 95% CI for *Enterococcus* isolates across One Health sample sources.

Sample Source	Antibiotic	Resistant n/N	% Resistant	95% CI
Chicken	E	21/25	84	65–94
Chicken	AMP	2/25	8	2–25
Chicken	CIP	2/25	12	4–30
Chicken	VAN, LZD, and TET	0/25	0	0–13
Env IN	E	13/26	50	32–68
Env IN	AMP	2/26	7.7	2–24
Env IN	CIP	7/26	26.9	14–46
Env IN	VAN, LZD, and TET	0/26	0	0–13
Env OUT	E	14/25	56	37–73
Env OUT	AMP	6/25	24	11–43
Env OUT	CIP	9/25	36	20–55
Env OUT	VAN, LZD	0/25	0	0–13
Env OUT	TET	2/25	8	2–25
Human	E	14/25	56	37–73
Human	AMP	3/25	12	4–30
Human	CIP	7/25	28	14–48
Human	VAN, LZD, and TET	0/25	0	0–13

n/N = Number of resistant isolates/total tested. 95% CI = 95% Confidence interval for proportion. AMP = Ampicillin, CIP = Ciprofloxacin, E = Erythromycin, LZD = Linezolid, TET = Tetracycline, VAN = Vancomycin.

### Antimicrobial susceptibility patterns of *Enterococcus* isolates

#### Overall resistance trends

Among the 101 *Enterococcus* isolates tested, erythromycin had the highest overall resistance at 61.4% (62/101), followed by ciprofloxacin (25.7%; 26/101), ampicillin (12.9%; 13/101), linezolid (5.9%; 6/101), and tetracycline (2.0%; 2/101) (Figure 4a). No resistance to vancomycin, linezolid, or tetracycline was detected, although the upper 95% CI limits suggested the potential for low-level resistance (0%–13%) (Table 2).

#### Variation by One Health source

Erythromycin resistance was highest in chicken isolates at 84% (95% CI: 65–94) and moderate in environmental and human isolates (50%–56%) (Figure 4b). Resistance to ampicillin and ciprofloxacin was comparatively low in chicken isolates (8%–12%) but notably higher in environmental isolates (AMP: 7.7%–24%; CIP: 27–36%) and human isolates (AMP: 12%; CIP: 28%) (Figures 4c and 4d; Table 2).

#### MDR

Overall, 6.9% (7/101) of *Enterococcus* isolates were MDR. The most common MDR pattern was the erythromycin–ampicillin–ciprofloxacin combination.

### Antimicrobials commonly used in chicken farming

#### Overview of AMU across farms

Among the 102 poultry farms assessed, 100 farmers reported using one or more classes of antimicrobials in their production systems. Only two small-scale farmers rearing indigenous chickens reported not using conventional antibiotics, instead using natural antimicrobial preparations.

#### Most frequently used antimicrobial classes

**Figure 5:**
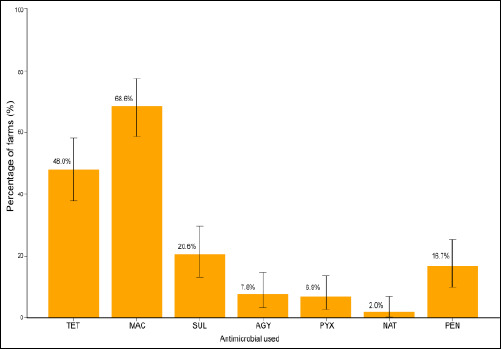
Commonly used antimicrobials in poultry farms in Kiambu County, shown as percentages with 95% confidence intervals. AGY = Aminoglycosides, MAC = Macrolide, NAT = Natural antimicrobial, PEN = Penicillin, PYX = Polymyxin, SUL = Sulfonamides, TET = Tetracycline.

Macrolides, tetracyclines, sulfonamides, and β-lactams were the most widely used antimicrobial classes among both large- and small-scale poultry producers in Kiambu County (Figure 5; Table 3). Macrolides were the most commonly used, reported by 68.6% of farmers (70/102). They were administered either individually or in combination with other antimicrobial classes.

**Table 3:** Percentage of antimicrobial use and 95% confidence intervals.

Antimicrobial used	Farms (n)	Percentage	95% CI (%)
Tetracycline	49	48	38–58
Macrolide	70	68.6	59–77
Sulfonamides	21	20.6	13–30
Aminoglycosides	8	7.8	3–15
Polymyxin	7	6.9	3–14
Natural antimicrobial	2	2	0.2–7
Penicillin	17	16.7	10–25

CI = Confidence interval.

#### Common antimicrobial brands and ingredients

Farmers frequently referenced commercial products containing erythromycin, a macrolide, including tylosin, tylodoxy, and aliseryl. Tetracycline-based antimicrobials were the second most commonly reported (48%; 49/102), followed by sulfonamides (20.6%; 21/102). The active ingredients of these products are detailed in Table 4.

**Table 4:** Common antibiotics and their active ingredients used by poultry farmers.

Antibiotic class	Trade name	Active ingredient (s)
Tetracyclines, macrolides, and polymyxin	Aliseryl^™^	Oxytetracycline, erythromycin, streptomycin, colistin, and vitamins
Tetracyclines, macrolides	Tylodoxy 200™	Doxycycline and tylosin
Macrolides	Tylosin^™^	Tylosin tartrate
Sulfonamides	Biotrim^™^, Biosol^™^	Trimethoprim–sulfamethoxazole
Aminoglycosides	Nemovit^™^	Neomycin
Penicilins	Ampicillin^™^	Ampicillin

Two poultry farmers used natural antimicrobials (effective microorganisms, EM-1).

### Patterns of AMU and phenotypic resistance

#### Associations between AMU and *E. coli* resistance

Analysis of farm-level AMU revealed several significant associations. Penicillin use was strongly correlated with ampicillin resistance in *E. coli*, with farms using penicillin having 32.9-fold higher odds of isolating ampicillin-resistant strains (OR = 32.95; 95% CI: 2.0–544.1; FDR_p < 0.001).In contrast, tetracycline use was not significantly associated with tetracycline resistance (OR = 0.90; 95% CI: 0.52–1.57; p = 0.78). Interestingly, sulfonamide use was associated with reduced odds of trimethoprim–sulfamethoxazole (SXT) resistance (OR = 0.46; 95% CI: 0.26–0.81; FDR_p = 0.015). Although polymyxin use yielded a high odds ratio for colistin resistance (OR = 101; 95% CI: 1.65–6175), the association was not statistically significant (p = 1), likely due to the small number of events (Table 5).

**Table 5:** Association between antimicrobial use at the farm-level and *Escherichia coli* isolate resistance.

AMU class	AMR	OR	95% CI	p-value	FDR_p	Significant after the FDR
AMU_TET	R_TE	0.9	0.52–1.57	0.777	1	No
AMU_PEN	R_AMP	32.95	2.0–544.06	0	0	Yes
AMU_SUL	R_SXT	0.46	0.26–0.81	0.0076	0.0153	Yes
AMU_AGY	R_CN	101	1.65–6175.23	1	1	No

AMU = antimicrobial use, AMR = Antimicrobial resistance, OR = Odds ratio calculated using 0.5 Haldane correction for zero counts, CI = Confidence interval, FDR = False discovery rate, FDR p-value: Benjamini–Hochberg correction to account for multiple comparisons. Significant after FDR: Yes, if FDR p < 0.05. R = resistance. AGY = Aminoglycosides, AMP = Ampicillin, CN = Gentamicin, PEN = Penicillin, SUL = Sulfonamides, SXT = Trimethoprim–sulfamethoxazole, TE = Tetracyclines, TET = Tetracycline.

#### Associations between AMU and *Enterococcus* resistance

Among *Enterococcus* isolates, tetracycline use was strongly associated with tetracycline resistance (OR = 20.2; 95% CI: 1.15–356.9; p = 0.0028), but significance was lost after FDR adjustment (FDR_p = 0.059). Penicillin use remained a significant predictor of ampicillin resistance after FDR correction (OR = 32.95; 95% CI: 1.99–544.06; FDR_p < 0.05). Similarly, sulfonamide use was associated with lower odds of SXT resistance (OR = 0.46; 95% CI: 0.26–0.81; FDR_p = 0.015), consistent with findings in *E. coli*. These associations remained significant following FDR adjustment (Table 6).

**Table 6:** Association between antimicrobial use at the farm-level and *Enterococcus* isolate resistance.

AMU class	AMR	OR	95% CI	p-value	FDR p-value	Significant after the FDR
AMU_TET	R_TET	20.22	1.15 – 356.91	0.0028	0.059	No
AMU_PEN	R_AMP	32.95	1.99–544.06	<0.001	<0.05	Yes
AMU_SUL	R_SXT	0.46	0.26–0.81	0.0076	0.015	Yes
AMU_AGY	R_CN	101	1.65–6175.23	1	1	No

AMU = antimicrobial use, AMR = Antimicrobial resistance, OR = Odds ratio calculated using 0.5 Haldane correction for zero counts, CI = Confidence interval, FDR = False discovery rate, FDR p-value: Benjamini–Hochberg correction to account for multiple comparisons. Significant after FDR: Yes, if FDR p < 0.05. R = resistance. AGY = Aminoglycosides, AMP = Ampicillin, CN = Gentamicin, PEN = Penicillin, SUL = Sulfonamides, SXT = Trimethoprim–sulfamethoxazole, TE = Tetracyclines, TET = Tetracycline.

## DISCUSSION

### MDR in *E. coli* and *Enterococcus* across One Health domains

This study documented notable MDR in *Escherichia coli* and *Enterococcus* isolates from human, animal, and environmental sources. In *E. coli*, resistance was observed across three key antimicrobial classes, penicillins, tetracyclines, and sulfonamides, with 35% of isolates exhibiting MDR. The most common MDR profile was ampicillin–tetracycline–trimethoprim–sulfamethoxazole. Comparable studies in East Africa have reported MDR *E. coli* prevalence of 52.2%, 53.7%, and 78.8% in humans, animals (including poultry), and environmental samples, respectively [22–24]. Among *Enterococcus* isolates, 6.9% exhibited MDR, consistent with previous reports of 86% MDR prevalence in poultry in Zambia [25] and 11% in humans in Tanzania [26].

### High ampicillin resistance and One Health implications

Across all sources, ampicillin resistance in *E. coli* was 77.2%, and notably, all isolates from chicken handlers’ hands (13/13) were resistant. This aligns with an Ethiopian One Health study in which human isolates showed higher resistance than animal isolates [27]. The widespread use of β-lactams in both human and veterinary medicine likely contributes to this elevated resistance [28, 29].

### Tetracycline and sulfonamide resistance in poultry systems

Tetracycline resistance was the second-highest resistance rate in *E. coli*, as expected given its affordability, broad-spectrum activity, and frequent use in livestock production [30]. Sulfonamide resistance (48%) also remained high, consistent with earlier findings from Kenya [31]. The popularity of sulfonamides for the management of bacterial and protozoal infections in poultry likely contributes to this trend [32].

### Resistance patterns in *Enterococcus* and public health risks

Among *Enterococcus* isolates, erythromycin resistance was highest (61%), followed by ciprofloxacin resistance (26%). These findings align with studies reporting high macrolide use and resistance in poultry production sectors across Africa [9, 33, 34]. Ciprofloxacin resistance across human–animal–environmental samples was highest in environmental isolates, followed by handlers and chickens, reflecting the public health risks associated with fluoroquinolone resistance in commensal bacteria [35].

### Environmental and human contributions to AMR transmission

Despite farmers reporting no fluoroquinolone use, ciprofloxacin resistance was observed, suggesting environmental contamination or human-to-animal transmission. Potential sources include contaminated water, farm equipment, shared hatcheries, cross-contamination by handlers, or circulation of resistant environmental strains. Further research is needed to elucidate specific transmission pathways among these One Health interfaces.

### Absence of vancomycin and carbapenem resistance

No vancomycin-resistant enterococci (VRE) isolates were detected, mirroring results from food-producing animals in Russia [36] and representing an encouraging finding given the clinical importance of vancomycin [37]. However, this contrasts with African meta-analyses reporting VRE prevalence of up to 26.8%, particularly in South Africa [38]. Likewise, carbapenem resistance was not detected in *E. coli*, although the reported use of polymyxins (colistin) on 7% of farms remains a concern given the drug’s role as a last-resort antimicrobial in human medicine [39]. Colistin resistance was not assessed, as CLSI guidelines recommend minimum inhibitory concentration (MIC) testing rather than disk diffusion.

### Anthropogenic drivers of AMR and environmental contamination

The association between AMU at the farm-level and phenotypic resistance supports the One Health concept that antimicrobial exposure generates selective pressure favoring resistant strains [40]. The extensive use of tetracyclines and sulfonamides on Kiambu poultry farms aligns with national reports [9] and corresponds with high resistance levels observed in *E. coli*. Tetracyclines, in particular, may enter the environment unmetabolized (40%–90% excreted unchanged), contributing to the persistence of AMR genes in soil and water systems [41, 42]. The presence of sulfonamide residues in Kenyan poultry products further underscores the potential for foodborne transmission [43]. Widespread AMR in Kenyan environments and animals increases the risk of human exposure [44], and sulfonamide resistance in Gram-negative bacteria is well documented [45].

### Macrolide use and persistence of resistance genes

No significant association was found between farm-level macrolide use and erythromycin resistance in *Enterococcus*. This is unsurprising, as macrolide resistance genes often reside on mobile genetic elements such as plasmids and transposons [46], enabling dissemination even in the absence of active macrolide use [4, 46]. Resistant strains or genes may be introduced through shared hatcheries, feed suppliers, handlers, water sources, or contaminated farm equipment [46–48]. Thus, current resistance patterns likely reflect historical antimicrobial practices, bacterial movement between farms, or co-selection driven by other antimicrobial classes.

### Importance of commensal bacteria in AMR surveillance

Overall, the findings highlight the crucial role of commensal bacteria such as *E. coli* and *Enterococcus* as indicators of AMR emergence across interconnected One Health environments. The coexistence of humans and poultry in shared spaces facilitates cross-contamination, undermining the efficacy of antimicrobial therapies for both human and animal health. To mitigate AMR, strict stewardship and regulatory measures governing critically important antimicrobials, including polymyxins and third- and fourth-generation cephalosporins, are required within poultry production systems.

## CONCLUSION

This study provides compelling evidence of substantial AMR across human, animal, and environmental components of poultry farming systems in Kiambu County, Kenya. High resistance to ampicillin, tetracycline, and trimethoprim–sulfamethoxazole in *E. coli*, coupled with notable resistance to erythromycin and ciprofloxacin in *Enterococcus* spp., underscores the interconnected nature of AMR transmission within the One Health continuum. Approximately 35% of *E. coli* and 6.9% of *Enterococcus* isolates exhibited MDR, and resistance profiles strongly reflected on-farm AMU patterns. The significant association between penicillin use and ampicillin resistance, and the reduced trimethoprim–sulfamethoxazole resistance in farms using sulfonamides, further illustrates the direct influence of antimicrobial practices on phenotypic resistance outcomes.

The practical implications of these findings are substantial. The widespread use of macrolides, tetracyclines, and sulfonamides on poultry farms, often without veterinary oversight, creates sustained selective pressure that accelerates the emergence and dissemination of AMR. The detection of resistant organisms on chicken handlers’ hands and in poultry environments underscores the ease with which resistant bacteria can move between animals and humans, posing risks to food safety, occupational health, and community-level transmission. Regulation of critically important antimicrobials, improved farm hygiene, and stronger antimicrobial stewardship programs are urgently needed.

A key strength of this study is its integrated One Health approach, examining AMR in humans, animals, and shared environments simultaneously using standardized CLSI methodologies. The use of archived isolates enabled a comprehensive examination of resistance profiles across multiple farm compartments. However, the study also has limitations, including the absence of molecular characterization of resistance genes, the lack of MIC testing for colistin, and reliance on self-reported AMU, which may be subject to underreporting or recall bias.

Future research should incorporate genomic AMR profiling, longitudinal monitoring of AMU and AMR trends, quantification of antimicrobial residues in poultry products, and evaluation of biosecurity interventions to more precisely trace transmission pathways. Expanding surveillance to additional counties and production systems would further strengthen national AMR mitigation strategies.

In conclusion, the study underscores that AMR in poultry farming is both a public health and an agricultural challenge. Coordinated One Health actions that link policy, surveillance, farm-level training, and responsible AMU are essential to curbing the spread of resistant bacteria and safeguarding the efficacy of lifesaving antimicrobials in both human and veterinary medicine.

## DATA AVAILABILITY

The data supporting this study are included in the manuscript.

## AUTHORS’ CONTRIBUTIONS

AKM: Conceptualization, methodology, validation, investigation, data collection, investigation, project administration, and writing original draft. PMM: Data analysis, visualization, validation, and manuscript drafting, reviewing, and editing. LCB: Conceptualization, methodology, supervision, and manuscript reviewing, and editing. CMM: Supervision and manuscript reviewing and editing. JMM: Conceptualization, methodology, validation, supervision, and manuscript reviewing and editing. All authors have read and approved the final version of the manuscript.
